# Emerging regenerative medicine and tissue engineering strategies for Parkinson’s disease

**DOI:** 10.1038/s41531-019-0105-5

**Published:** 2020-01-08

**Authors:** James P. Harris, Justin C. Burrell, Laura A. Struzyna, H. Isaac Chen, Mijail D. Serruya, John A. Wolf, John E. Duda, D. Kacy Cullen

**Affiliations:** 1 Center for Brain Injury & Repair, Department of Neurosurgery, Perelman School of Medicine, University of Pennsylvania, Philadelphia, PA USA; 2 Center for Neurotrauma, Neurodegeneration & Restoration, Corporal Michael J. Crescenz Veterans Affairs Medical Center, Philadelphia, PA USA; 3 Department of Bioengineering, School of Engineering and Applied Science, University of Pennsylvania, Philadelphia, PA USA; 4 Department of Neurology, Thomas Jefferson University, Philadelphia, PA USA; 5 Department of Neurology, Perelman School of Medicine, University of Pennsylvania, Philadelphia, PA USA; 6 Parkinson’s Disease Research, Education, and Clinical Center (PADRECC), Michael J. Crescenz Veterans Affairs Medical Center, Philadelphia, PA USA

**Keywords:** Neurodegeneration, Basal ganglia, Regenerative medicine, Regeneration and repair in the nervous system, Parkinson's disease

## Abstract

Parkinson’s disease (PD) is the second most common progressive neurodegenerative disease, affecting 1–2% of people over 65. The classic motor symptoms of PD result from selective degeneration of dopaminergic neurons in the substantia nigra pars compacta (SNpc), resulting in a loss of their long axonal projections to the striatum. Current treatment strategies such as dopamine replacement and deep brain stimulation (DBS) can only minimize the symptoms of nigrostriatal degeneration, not directly replace the lost pathway. Regenerative medicine-based solutions are being aggressively pursued with the goal of restoring dopamine levels in the striatum, with several emerging techniques attempting to reconstruct the entire nigrostriatal pathway—a key goal to recreate feedback pathways to ensure proper dopamine regulation. Although many pharmacological, genetic, and optogenetic treatments are being developed, this article focuses on the evolution of transplant therapies for the treatment of PD, including fetal grafts, cell-based implants, and more recent tissue-engineered constructs. Attention is given to cell/tissue sources, efficacy to date, and future challenges that must be overcome to enable robust translation into clinical use. Emerging regenerative medicine therapies are being developed using neurons derived from autologous stem cells, enabling the construction of patient-specific constructs tailored to their particular extent of degeneration. In the upcoming era of restorative neurosurgery, such constructs may directly replace SNpc neurons, restore axon-based dopaminergic inputs to the striatum, and ameliorate motor deficits. These solutions may provide a transformative and scalable solution to permanently replace lost neuroanatomy and improve the lives of millions of people afflicted by PD.

## Overview: regenerative medicine

The field of regenerative medicine encompasses the use of cell replacement strategies and tissue engineering to promote regeneration and functional restoration following injury or disease.^1^ Cell delivery strategies may replace lost cells in cases where endogenous cells are insufficient or dysfunctional (e.g., new neurons). Tissue engineering techniques generally combine aspects of biomaterial scaffolds and cell replacement techniques to create a three-dimensional (3-D) environment to influence cell (native and/or implant) behavior such as phenotype, architecture, migration, and survival. Indeed, biomaterials can provide 3-D structure for host cell infiltration, differentiation, and organization, and may also serve as a means for drug administration (e.g., controlled release). In the central nervous system (CNS), both cell replacement and tissue engineering strategies are being vigorously pursued to facilitate regeneration of native tissue and/or to directly restore lost function based on permanent structural integration.^2–5^ Although the primary long-term goal for the treatment of Parkinson’s disease (PD) is to develop a means to arrest the progressive neurodegenerative pathology, the objective of the field of regenerative medicine is to advance restorative treatments to functionally replace and/or reconstruct neuronal circuitry that has succumbed to the disease. This article reviews the historic, current, and emerging regenerative medicine strategies for PD, emphasizing the importance of reconstructing the entire nigrostriatal pathway for consistent and durable recovery of function. Further, we will discuss the challenges associated with translating these solutions into clinical practice.

## Parkinson’s disease

### Epidemiology and societal burden

PD is a progressive neurodegenerative disease that causes significant morbidity across a prolonged and progressive disease course. PD is characterized by resting tremor, bradykinesia (slowness of movement), rigidity, and other symptoms that decrease quality of life, ultimately leading to significant disability via the inability to control motor function.^6,7^ In the United States, 50,000–60,000 cases are diagnosed each year with a prevalence of over one million people.^7^ As the disease affects 1–2% of people over 65, its prevalence is expected to double in the next 20 years.^8^ The cost of PD is estimated to be ~$35 billion per year in the United States alone.^9^ Recent studies have estimated that arresting PD progression would result in net monetary benefits of almost $450,000 per patient, and if PD progression was slowed by 20%, a benefit of over $75,000 per patient would be realized.^9^

### Pathophysiology and neuropathology

**Fig. 1:**
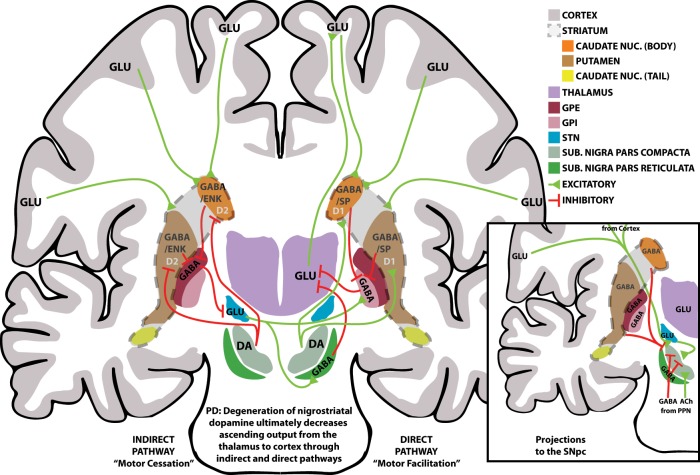
Overview of basal ganglia neuroanatomy. A schematic overview of the primary motor circuits in the basal ganglia, the indirect (left) and direct (right) pathways. Note, pathways crossing sides does not imply decussation, rather the contralateral connections separate the indirect and direct pathways. Excitatory connections are depicted in green with triangle ends, inhibitory connections are depicted in red with “T” ends. Not all connections are depicted, including but not limited to, all connections from thalamus to cortex, all connections from cortex to striatum, connections to/from caudate nucleus (tail), connections from cortex to brainstem, and inputs to SNpc (pictured in the inset). Inset: a schematic overview of inputs to SNpc found in literature. The PPN is located caudal to the substantia nigra and inputs are depicted as such. Inputs from the caudate nucleus (tail) is not pictured, and not all inputs from the cortex are depicted. Merging of signals from the cortex or caudate/putamen are done for illustrative purposes. D1; D1 receptors; D2; D2 receptors; DA dopamine; ENK enkephalin; GLU glutamate; NUC nucleus; PPN pedunculopontine nucleus; SP substance P; SUB substantia.

Motor symptoms of PD are caused by the selective loss of dopaminergic neurons in the substania nigra pars compacta (SNpc) projecting to the dorsal striatum, which is composed of the caudate and putamen (see Fig. 1 for an overview of the relevant neuroanatomy). SNpc degeneration is thought to result from the formation of Lewy bodies and Lewy neurites, inclusions in cytoplasm and processes, respectively.^10^ The primary components of Lewy pathology are filaments of α-synuclein, a neuronal protein normally found in synaptic terminals.^11^ Lewy pathology also occurs outside of the SNpc and striatum, including in the olfactory bulb, medulla, pons, spinal cord, and peripheral autonomic system, and in later stages of PD, the midbrain, amygdala, hippocampus, and several other cortical regions.^12,13^ At the time of motor symptom onset, at least 60% of SNpc cells have degenerated.^10^ There are several theories as to why dopaminergic neurons are preferentially impacted, but many believe that a combination of factors including structure, function, and metabolic needs make dopaminergic neurons particularly vulnerable to the pathophysiology of PD.^14^

Non-motor symptoms associated with PD affect nearly the entire body, encompassing a wide range of manifestations including sensory deficits and pain, irregular sleep cycle patterns and dream re-enactment behavior, cognitive decline, depression, anxiety, apathy, psychosis, urinary dysfunction, constipation, and other autonomic dysfunctions.^15,16^ Although PD is commonly associated with motor symptoms, some non-motor symptoms, such as olfactory dysfunction and dream re-enactment behavior, can present before the onset of the classic motor symptoms of PD, whereas others manifest at later stages, such as cognitive decline and neuropsychiatric disorders.^17^ Widespread α-synuclein pathology is often observed throughout the nervous system in regions associated with the manifestation of non-motor symptoms, however the exact mechanisms remain unclear.^18^ Growing evidence has implicated neurotransmitter depletion (e.g., noradrenergic, serotonergic, and cholinergic) in multiple interconnected CNS pathways, specifically between the striatum, prefrontal cortex, limbic system, and spinal cord, in the manifestation of many non-motor symptoms of PD.^19^

### Importance of the nigrostriatal pathway and implications of its loss

As SNpc neurons send long axonal projections to the striatum, the stereotypical neurodegeneration that occurs in PD deprives the striatum of crucial dopaminergic inputs and thereby interrupts important motor feedback pathways. The nigrostriatal pathway is a major component of the basal ganglia, the interconnected structures within the brain involved in motor control (Fig. 1). The main structures in the basal ganglia circuitry are the dorsal striatum (caudate nucleus and putamen), the core of the nucleus accumbens, the globus pallidus, the subthalamic nucleus (STN), and the substantia nigra. The primary input target of the basal ganglia is the striatum, which integrates the incoming sensorimotor information via projections from the neocortex, intralaminar nuclei of the thalamus, and midline nuclei of the midbrain. Output projections from the internal segment of the globus pallidus (GPi) and pars reticulata of the substantia nigra (SNpr) are modulated by two basal ganglionic circuits, which are referred to as the “direct” and “indirect” pathways.^20^ The direct pathway serves to increase movement with inhibitory projections from the striatum to the SNpr and GPi. The indirect pathway serves to decrease movement with inhibitory projections to the external segment of the globus pallidus (GPe), which in turn sends inhibitory projections to the STN that sends excitatory projections to the GPi and SNpr.^21^ These pathways are antagonistic: the direct pathway leads to motor facilitation, and the indirect pathway suppresses unwanted motor movement (motor cessation).

As such, a main component of the basal ganglia motor circuit is the nigrostriatal pathway with dopaminergic projections originating from neurons in the SNpc. The projections comprise the highly dense axonal arborization found mostly in the striatum with some collateral arborization in the GPe. Dopamine-releasing axons in the nigrostriatal pathway synapse onto GABAergic medium spiny neurons in the striatum. In the direct pathway, the terminals synapse to D1 receptors, and in the indirect pathway, the terminals synapse to D2 receptors. Dopamine release activates the direct D1-receptor-mediated pathway that ultimately facilitates motor movement. In non-pathologic conditions, the cortex activates the indirect pathway with a smaller contribution of inhibition through SNpc dopamine release on D2-receptor cells in the striatum. In PD, the degeneration of SNpc dopamine neurons shifts the balance of direct and indirect pathways. The net result of both pathways is inhibitory cells in the GPi/SNpr are more active to create a stronger inhibition of the thalamus that culminates in less activation of the motor cortex.

The diagram of the indirect and direct pathways (Fig. 1) is meant to approximate the primary pathways for motor movement, and includes an insert containing an important aspect often left out of published pathway diagrams: input to the SNpc, which modulate basal ganglia activity, thus closing the feedback loop (Fig. 1 insert). Indeed, the literature indicates the presence of a wide array of inputs into the SNpc including GABAergic from the caudate/putamen, glutamatergic from the cortex and STN, glutamatergic and cholinergic from pedunculopontine nucleus, and GABAergic input from the SNpr.^22–25^ It is probable that these inputs to the SNpc are vital for highly functioning motor feedback circuits.^26,27^

The striatum is further organized into two distinct compartments comprised of dendrite and local axon collaterals known as the striosomes and the matrix.^28^ The matrix compartment relays information from sensorimotor input and output neurons to the GPi and SNpr via the direct and indirect pathways. Striosomes, also known as patch compartments, are widely distributed regions within the striatum comprising ~15% of the volume in the striatum. The striosome compartments form an interconnected 3-D labyrinthine network within the striatum. Striosome neurons are thought to modulate the entire dorsal striatum via the inhibitory projections to the dopaminergic neurons in the SNpc (not pictured in Fig. 1).

**Fig. 2:**
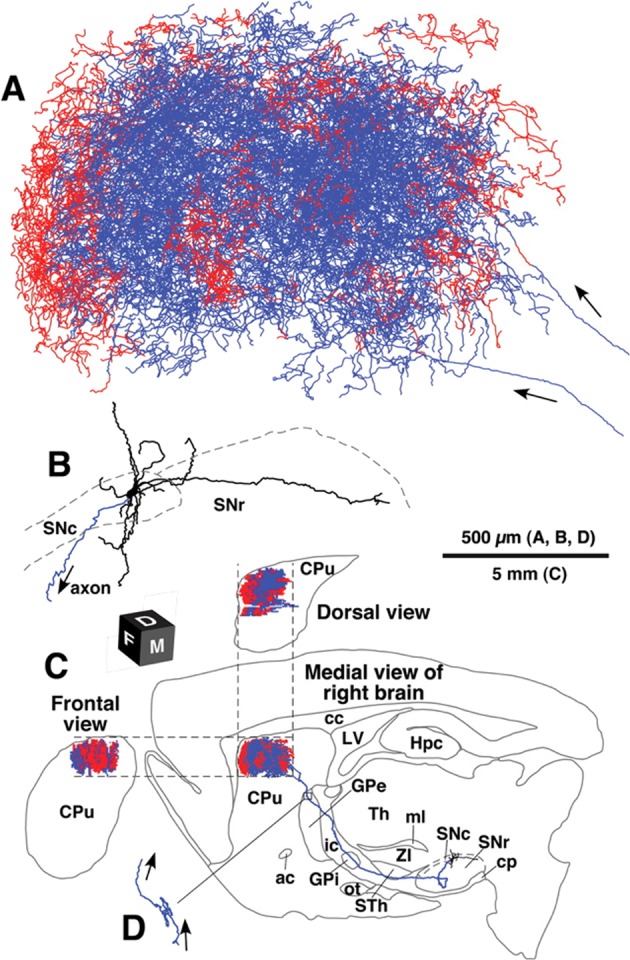
Rich dopaminergic axon arborization in the striatum. Camera lucida reconstruction of a dorsal SNpc neuron. **a** The axon fibers in the striatum and **b** dendrites in the SNpc were projected onto a parasagittal plane and superimposed from the medial side. **c** The dorsal and frontal views of the intrastriatal axonal arborization were reconstructed and compared with the medial view. Red and blue lines in the striatum indicate the axon fibers located in the striosome and matrix compartments, respectively. Red fibers at the most rostral portion in **a** were mostly located in the MOR-positive subcallosal streak. **d** The axon gave rise to only minor collaterals in the external segment of the GPe. ac anterior commissure; cc corpus callosum; cp cerebral peduncle; CPu caudate–putamen (neostriatum); Hpc hippocampus; ic internal capsule; LV lateral ventricle; ml medial lemniscus; MOR μ-opioid receptor; ot optic tract; SNc substantia nigra pars compacta; SNr substantia nigra pars reticulate; STh subthalamic nucleus; str superior thalamic radiation; Th thalamus; ZI zona incerta. (Image and caption adapted from Matsuda et al.^28^ and reprinted with permission from publisher).

Long-projecting axons from SNpc neurons exhibit an exquisite arbor in the striatum to release dopamine at highly dense varicosities along the axon, which are organized *en passant* with neighboring neuronal compartments (Fig. 2). Dopamine has a rapid half-life, which limits its sphere of influence. However, the highly dense arborization of dopaminergic neurons provides overlapping and redundant innervation of the striatum, which leads to increased temporal and spatial effects compared with what can be achieved from a single dopaminergic neuron.^29^ Detailed anatomical work in rodents showed that a single dopaminergic neuron innervates, on average, 2.7% of the neurons in one striatum (e.g., ~75,000 out of 2,790,000 striatal neurons in one hemisphere of the rodent brain).^28^ Moreover, because of the redundant overlapping arborization, it is estimated that a single striatal neuron is under the influence of 95–194 dopaminergic neurons from the SNpc. Consequently, this highly redundant organization might contribute to the clinical presentation of PD; patients only develop symptoms of Parkinsonism following an extensive loss of dopaminergic neurons (>60%).^28–30^ The complexity and nuances of the basal ganglia circuitry and the widespread innervation of a single nigrostriatal axon, let alone all nigrostriatal inputs, warrant thoughtful consideration regarding techniques for adequate circuit reconstruction.

## Historic and current treatments for PD

### Evolution of care: pharmacological interventions to neuromodulation

As far back as the 1940s, the advent of the stereotactic head frame allowed the targeting of subcortical structures with great precision.^31^ Surgeons discovered that ablation or lesion therapy could modulate brain activity and ameliorate the symptoms of PD.^32^ However, the discovery of levodopa (or other dopamine agonists) led to a shift away from surgical therapy to chronic pharmaceutical treatment.^33^ These agents attempt to compensate for the underproduction of dopamine owing to cell loss. In the early 1990s, Laitinen et al. reintroduced the posteroventral pallidotomy and reported significant improvements for all motor complications of PD.^34^ During pre-thalamotomy mapping studies, high-frequency electrical stimulation ameliorated PD symptoms, leading to the development of DBS.^35^ By the mid-1990s, DBS of the GPi and STN was also shown to be effective. Indeed, in appropriately selected patients where levodopa-related complications are disabling, modulation of brain function via DBS of the GPi or STN has been shown to be superior to continued medical management.^32^ In many cases, pharmacological treatments are the first step in treatment, followed by DBS as a second step. Often, DBS patients continue levodopa or other pharmacological treatments, although often at a lower dose.

Although these treatments have led to significant improvements in movement disabilities and improved the quality of life for patients with PD, they are treating symptoms resulting from the loss of dopaminergic input to the striatum rather than resolving the underlying neurodegeneration. In addition, there are often unwanted side effects. For instance, DBS stimulation may detrimentally impact cognition and speech.^36^ Moreover, any current treatment regimen—pharmaceutical management, DBS, or a combination of the two—often fails to give the same clinical benefit over time. The lack of long-term efficacy is generally attributed to one of two issues: (1) the advent of adverse side effects (e.g., dyskinesias, motor fluctuations, behavioral addictions, and/or impulse control disorders),^37^ or (2) the ongoing degeneration of nigrostriatal neurons decreasing clinical benefits. As such, even with state-of-the-art management, the average duration from minimal disability to confinement to bed or wheelchair is ~13 years.^9^

New pharmaceutical therapies that focus on neuroprotection are being developed, although to date, there are no proven therapies to slow progression of disease.^38^ In addition, these treatments would not restore innervation to the striatum, as they would not replace SNpc dopaminergic neurons that had degenerated before the onset of motor symptoms.^10^ Therefore, a method to diagnose PD pathology before the onset of motor symptoms would be necessary for these pharmacological methods to maintain motor function.^39^ Although there is growing evidence that non-motor symptoms have a key impact on quality of life, only a few large randomized clinical trials have focused on management of non-motor symptoms.^16^ Dopaminergic replacement strategies are ineffective for treatment of most of the non-motor symptoms, and are commonly associated with non-motor side effects including somnolence, orthostatic hypotension, visual hallucinations, and nausea.^40^

Gene therapies to relieve PD symptoms have recently reached clinical trials evaluating the efficacy and safety of delivering vectors with genes encoding for overexpression of (1) glutamic acid decarboxylase in the subthalamic nuclei to increase the GABA basal tone,^41,42^ (2) enzymes to increase endogenous striatal dopamine synthesis or conversion of l-dopa to dopamine,^43^ and (3) neurturin—a ligand similar to glial cell line-derived neurotrophic factor (GDNF) that has been shown to enhance survival and outgrowth of dopaminergic neurons.^44,45^ To date, out of these gene therapy clinical trials, only the vector with the gene encoding for increased production of glutamic acid decarboxylase has demonstrated beneficial motor function.^46^ Therefore, although gene therapy might be a promising strategy, development of an effective treatment requires more research.

### Cell transplant-based treatments

Prior to the 1970s, it was widely believed that restorative treatments for neurodegenerative diseases of the CNS were not possible. However, two articles published in the same year demonstrated that fetal mesencephalic grafts rich in dopaminergic neurons could ameliorate Parkinsonism symptoms in rodent models.^47,48^ Since then, many studies have examined the potential of cell transplant-based treatments to restore dopamine in the striatum and ameliorate motor deficits of PD.^49^ As of 2004, over 350 patients had received cell replacement therapies, and in many cases, patients were tapered off drugs and witnessed a decrease in motor symptoms.^50^ Successful fetal tissue grafts have survived over two decades in some patients despite ongoing PD pathology.^51^ Indeed, long-term survival of grafted dopaminergic neurons with extensive putamenal dopaminergic innervation was reported in PD patients at postmortem.^51,52^ In addition, ^18^F-fluorodopa imaging demonstrated robust dopaminergic uptake within the striatum. Clinical trials have indicated beneficial results from cellular grafts when methods sustain a novel population of at least 80,000 dopaminergic neurons, although, in a recent study, motor benefits were observed with only 40,000 neurons surviving after 24 years.^52,53^

Although inconsistent, clinical improvements, such as decreased motor symptoms, were observed in some cases; however, widespread clinical adoption of tissues grafts has been stymied by several notable limitations. A subset of patients had adverse side effects or no significant improvements.^50^ Moreover, grafts were sourced from fetal tissue, and the variability of source tissue likely contributed to some trials showing limited to no efficacy. In addition, multiple fetal donors (typically 3–5) must be pooled to source a sufficient number of cells for one patient, which may contribute to the heterogeneity of outcomes and likely indicates a lack of material for widespread clinical usage. This is exacerbated by ethical concerns associated with fetal tissue that make access difficult in some instances, thereby limiting procedures. Also, trials have had varied preparation, storage, and immunosuppression regimes that may have affected the health of the grafts, again creating a source of variability and likely affecting efficacy. Thus, fetal grafts have inherent issues with consistency and requirements for immunosuppression.^54^

Another concern is that the majority of these studies involved cellular graft implantations in the striatum, not the SNpc—the location of dopaminergic neurons naturally providing input to the striatum. Whereas cells implanted into the striatum may create new “factories” for dopamine, these cells do not receive their normal inputs to control their activity, potentially resulting in dysregulated dopamine release and related side effects. As discussed previously, the SNpc receives a wide range of inputs, and these inputs are thought to be important for restoring the full motor circuit.^26,27^ The tradeoffs between different options for placement of cells/grafts is covered in more depth below, but complete function of the motor control system relies on well-controlled feedback loops that fine-tune dopamine levels. Although significant progress has been made, fetal tissue grafts do not appear to present a viable or sustainable strategy to repair the nigrostriatal pathway and replicate both the outputs and inputs for dopamine regulation to the striatum from the SNpc. However, success of fetal grafts in PD patients demonstrates the potential for dopaminergic neuron transplants to provide long-term benefits, provided there are improvements in cell/tissue supply, consistency, and ideally, actual recreation of the nigrostriatal pathway.

## Regenerative medicine approaches to treating PD

### The challenge of CNS regeneration

The extraordinary computational capabilities of the human brain rely on vast axonal connections spanning long distances that form sophisticated neural circuits and enable profound parallel processing, often referred to as the connectome.^55^ Degeneration and disconnection of these axonal pathways as well as localized neuronal degeneration frequently occur in many CNS disorders, including traumatic injury, stroke, PD, and others.^56^ Unfortunately, functional regeneration rarely occurs in the CNS and neurogenesis is restricted to a few distinct domains, such as the subventricular zone and the dentate gyrus of the hippocampal formation. In the majority of the CNS, natural regeneration of long axon pathways does not occur, mainly owing to endogenous inhibition of axon growth, absence of directed guidance to far distant targets, and loss of intrinsic capacity for long outgrowth in mature neurons. The lack of neurogenesis and correctly targeted axonal regrowth are key limitations in endogenous CNS regeneration and repair, thereby resulting in diminished recovery and continued functional deficits.

### Regenerative medicine

The field of regenerative medicine is pursuing novel approaches to develop cellular and tissue constructs to facilitate regeneration and/or restore function following injury, aging, or disease. These techniques are being applied to address the limitations in repair and regeneration in the CNS, and in the particular case of PD, aim to build upon the benefits of cellular grafts seen in human patients. Using novel biomaterials, tissue culture techniques, and knowledge from previous experiments, the concerns associated with cellular grafts, such as consistency of cells, need for immunosuppression, placement of cells, and source of tissue (supply and ethics), can be addressed.

### Design goals to restore the nigrostriatal pathway

The pioneering work from the 1970s and 1980s demonstrated the potential for allografted fetal tissue to improve striatal dopamine levels in some patients with PD. These studies also paved the way to design criteria for cell-based therapies prior to clinical trials including:

- Long-term survival of dopaminergic neurons into the host striatum with robust fiber outgrowth in the adult rodent brain.^57^
- Afferent and efferent synaptic integration with the host rodent brain (i.e., receiving local inputs from the host brain and forming connections with host striatal end target) following implant of dopaminergic neurons into a cortical cavity.^58,59^
- Adequate dopamine release in a controlled fashion following intrastriatal grafts in adult rats.^60^
- Amelioration of motor deficits in rodent models of PD following intracerebral grafting of dopaminergic neurons.^61^

To actually recapitulate the native anatomy of the nigrostriatal pathway would call for grafts, originating in the substantia nigra and terminating in the striatum. Initially, for proof-of-concept studies in rats, the literature suggests a requirement of ≥1200 morphologically healthy dopaminergic neurons with a unidirectional axonal architecture and ≥5–6 mm in length that result in dopamine release in the range of 50–100 nm in the striatum for functional benefits to be seen.^62,63^ Based on human fetal graft studies, these numbers translate to 40,000–80,000 healthy dopaminergic neurons, unidirectional axonal architecture of ≥3–5 cm in length, and dopamine release of ≥7 ng/mg of tissue for nigrostriatal pathway restoration in humans (Table 1).^53,64^ In addition, owing to post transplant attrition, pre-transplant constructs will likely require a greater number of dopaminergic neurons (e.g., ≥2400 healthy dopaminergic neurons with 50% survival would result in ≥1200 neurons), although the exact rates of attrition would require further research.

**Table 1:** Target dopaminergic neuron densities and dopamine production.

	Rat	Human
No. of dopaminergic neurons required:		
Dopamine cell therapeutic threshold	≥1200–2400^a^	≥40,000–80,000^b^
Therapeutic dopamine production	50–100 nm^c^	7 ng/mg of tissue^d^

^a^Data from Isacson, Bjorklund^63^

^b^Data from Bjorklund and Lindvall^53^

^c^Data from Robinson, Venton et al.^62^

^d^Data from Kish, Kalasinsky et al.^64^

**Fig. 3:**
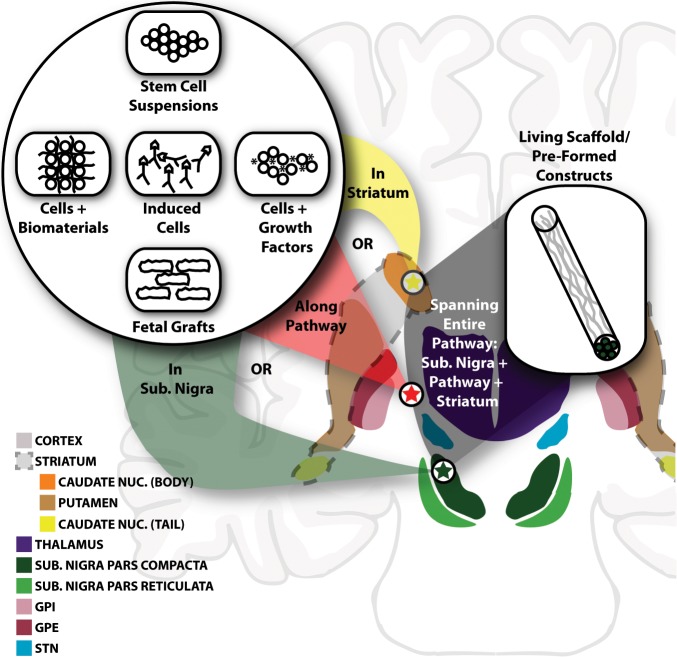
Overview of regenerative medicine-based repair strategies for PD. A schematic overview of the possible repair strategies for the nigrostriatal pathway. The figure depicts the same circuit diagram schematic shown in Fig. 1 with circuit connections removed. The top left inset includes the various cell sources used (Consideration 1). The red, green, and yellow arrows indicate the various locations that cells have be implanted (Consideration 2). The inset to the right shows a preformed construct solution to restore the nigrostriatal pathway, including dopaminergic cell bodies in the SNpc, the inputs to those dopaminergic cells from other neural structures, and their long axonal outputs to the striatum. NUC nucleus; SUB substantia.

To sidestep issues with tissue grafts, researchers have studied myriad cell-based therapies to treat PD.^65^ Cell replacement strategies use endogenous or exogenous cell sources, including stem cells, and are similar in concept to the tissue graft techniques but generally involve cell dissociation and/or expansion.^4^ The usage of stem cells allows for therapies that can include secretion of neuroprotective factors as well as self-renewing cells that give rise to additional neurons or other cell types.^3^ To restore the entire nigrostriatal pathway with appropriate inputs and outputs remains a considerable challenge, as implantation of only dissociated cells cannot restore the key anatomic features of damaged pathways—notably long axon tracts projecting to proper anatomical structures. Key considerations include (1) cell source, (2) location of the transplant, and (3) strategy to facilitate sufficient and appropriate axonal outgrowth to the striatum (summarized in Fig. 3).

For cell replacement strategies that aim to restore the entire nigrostriatal pathway, the first key consideration is the cell source of the graft material. The development of the 6-hydroxydopamine (6-OHDA)-lesioned rodent model of PD, which selectively and irreversibly degenerates dopaminergic neurons in the lesioned nigrostriatal pathway, has led to various approaches to determine whether restoration of dopaminergic tone could ameliorate Parkinsonian motor symptoms.^47,48^ Moreover, several grafting strategies using different dopamine-producing tissues have been evaluated in clinical trials, such as adrenal medullary cells (AMCs), sympathetic ganglia, carotid body cells, retinal pigmentary epithelial cells linked to microcarriers, and porcine ventral mesencephalon tissue.^66^ However, the most-effective transplantation strategy has been with human fetal mesencephalic tissue. Preclinical rodent studies have shown that transplanted dopaminergic neurons from human fetal mesencephalic grafts can innervate the dopamine-deprived striatum, receive inputs from host neurons, and diminish rotational behavioral symptoms.^67,68^ These developments led to clinical trials using human ventral mesencephalic tissue that demonstrated survival of grafted dopaminergic neurons up to 24 years after transplantation, integration with the brain circuitry, and improvement in motor symptoms.^51,52,69–72^ Despite these promising findings, clinical improvement has varied across different trials likely owing to inadequate standardization of patient enrollment, tissue harvest, graft location, surgical implantation, and immunosuppression.^73^ Although the early preclinical experience and clinical successes using fetal grafts has been crucial to establish the potential for a cell transplantation strategy in PD, further developments have been challenging owing to ethical concerns and limited tissue availability.

Alternative, non-fetal cell source candidates have been developed to avoid these concerns with the goal of attaining the same outcomes as the early fetal transplantation studies. Non-neuronal, catecholamine-producing cell sources, such as AMCs and retinal pigmentary epithelial cells, have been considered as a potential alternative to fetal grafts by producing and secreting dopamine in the striatum, thereby restoring motor function.^49^ However, these grafts did not demonstrate the same potential to restore the dopamine levels in the striatum and ameliorate behavioral deficits as the fetal grafts in preclinical studies; therefore, it was not surprising that clinical trials evaluating non-neuronal cell sources did not result in significant motor improvements compared with the relatively successful human fetal ventral mesencephalic grafts.^66^ These experiments illustrate the importance of validating potential grafting strategies by demonstrating robust cell survival, host integration, and dopamine release in animal models before clinical trials.

Building on the knowledge gained from transplantation studies using fetal grafts or non-neuronal cell types, it has become clear that a successful grafting strategy likely requires use of a cell source capable of generating authentic dopaminergic neurons that resemble those found in the SNpc and can re-establish lost striatal innervation.^74^ Indeed, various approaches have been developed to generate dopaminergic-like neurons from alternative cell sources, such as embryonic stem cells (ESCs), induced pluripotent stem cells (iPSCs), expanded neural precursor cells (NPCs), mesenchymal stem cells (MSC), and direct neuronal reprogramming.^49^

Pluripotent stem cells are a promising alternative to fetal ventral mesencephalon grafts owing to their capability to provide a potentially unlimited source of any cell type. Advancements in the fields of stem cell neurobiology have led to the generation of two different types of pluripotent stem cells: (1) ESCs derived from the inner cell mass of early-stage embryos and (2) iPSCs derived from de-differentiated somatic cells. Early studies demonstrated that ESC-derived dopaminergic neurons were capable of surviving in an adult rodent brain, however, incomplete differentiation led to increased risk of tumor formation and neural overgrowth.^75^ Recent studies appear to have resolved these issues with improved differentiation protocols and cell culture techniques.^76,77^ However, despite these advancements, widespread development of ESC-derived dopaminergic cell therapies has remained limited for the same inherent reasons as fetal transplantations; lack of tissue availability and ethical concerns.

The discovery of somatic cell de-differentiation has led to increased interest in iPSCs as a promising alternative cell source for future PD therapies owing to the potential of generating patient-specific iPSC-derived dopaminergic neurons.^65^ Although early preclinical data did not demonstrate similar outgrowth or functional restoration in rodent and non-human primate (NHP) models of PD compared with fetal grafts, advancements in developmental neurobiology led to the discovery that dopaminergic neurons are derived from floor plate cells, not neuroepithelial progenitors like every other neuron in the brain.^78^ This new finding enabled the development of novel molecular differentiation techniques for generating more “authentic-like” dopaminergic neurons resembling those found in the SNpc.^79^ Furthermore, human ESC- and iPSC-derived dopaminergic neurons generated with these refined differentiation protocols seem to have solved the earlier issues with tumorgenicity, and enabled long-distance targeted striatal innervation.^76,77,80^ Although iPSC-derived neurons have been shown to survive transplantation, variable amounts of neurite outgrowth has been reported, which is likely responsible for inconsistent functional restoration.^81^

Neural stem cells (NSCs) expanded from ESCs and iPSCs have been investigated as an alternative source for neural cell replacement treatment for PD owing to their multipotent and self-renewing properties.^82^ Unlike ESCs and iPSCs, NSCs have limited differentiation capability and are fated towards a brain cell phenotype, reducing the likelihood for tumor growth.^83^ NSCs have been shown to be an effective neural repair strategy in preclinical models of injury owing to their inherent ability to provide neuroprotection, decrease immunoreactivity, and secrete neurotrophic factors, such as glial-derived neurotrophic factor and brain-derived neurotrophic factor.^84^ Previous work has also shown that NSCs can differentiate into dopaminergic-like cells, survive transplantation, integrate with the host architecture, and improve functional recovery.^85^ Another promising direction is the development of an autologous cell source for transplantation using patient-specific iPSC-derived NSCs.^82^ Although intriguing, expansion of NSCs has remained challenging and likely requires further refinement for clinical trials. Moreover, further safety and efficacy preclinical studies are necessary to reduce the likelihood for graft and/or differentiation failure.^86^

MSCs are multipotent, non-hematopoietic stem cells capable of self-renewal that typically arise from bone marrow and differentiate into adipocytes, osteoblasts, and chondroblasts, and have been reported to elicit an anti-inflammatory effect.^87^ Elevated neuroinflammatory responses have been implicated in neurodegenerative diseases, such as PD and Alzheimer’s disease.^88^ These findings have led to increased interest in MSCs as a potential cell source candidate owing to their immunomodulatory effect.^89^ Indeed, MSCs have demonstrated the capability to differentiate into astrocyte-like cells that secrete neurotrophic factors and ameliorate the motor deficits in a rodent model of PD.^90^ In addition to immunomodulation, MSCs or MSCs differentiated into a neuron-like phenotype have been reported to migrate across the corpus callosum to a 6-OHDA-lesioned SNpc and striatum when initially transplanted in the contralateral hemisphere.^91^ However, it is unclear whether the cells are attracted to the lesion or the acute inflammation at the injection site.^92^

Direct neuronal reprogramming has recently become an interesting cell source candidate due to the potential for generating dopaminergic neurons from somatic cells by adding transcription factors for cell lineage conversion.^93^ This strategy could theoretically provide autologous dopaminergic neurons reprogrammed from a patient’s own somatic cells, alleviating the ethical and limited tissue availability with fetal cells and minimizing complications associated with allogenic transplants. Although reprogramming has historically used viral vectors, recent advancements in microRNA technology has led to the development of alternative transcription factor delivery methods that have been reported to increase the conversion efficiency in vitro and in vivo from somatic cells into functional dopaminergic neurons and ameliorate motor symptoms in a model of PD.^94^

The next key consideration for development of a successful regenerative strategy is the location of the graft. Based on the success of the early studies, several graft locations have been investigated, such as (1) in the striatum, (2) in the SNPc, or (3) along the entire length of the nigrostriatal pathway. Many cell-based transplantation strategies have primarily focused on using intrastriatal dopaminergic grafts to restore the physiological levels of dopamine within the striatum.^53^ As noted above, cells implanted into the striatum may create new “factories” for dopamine, but the cells do not receive the full suite of normal inputs that regulate dopaminergic production and delivery in the striatum. However, as striatal innervation is a critical requirement for any potential cell source, “short-circuiting” the nigrostriatal pathway is useful for developing effective stem cell therapies.

Intrastriatal transplantation of dopaminergic neurons in a NHP model of Parkinsonism has been reported to demonstrate extensive neurite outgrowth in the striatum and increased dopamine uptake near the graft, resulting in significant amelioration of the cardinal motor symptoms of PD.^95–99^ Despite these improvements, inconsistent functional recovery in animal models and human studies has been reported, likely owing to the variations in graft composition, low dopaminergic neuron survival, poor neurite extension, minimal graft volume, differing immunosuppressant regimes, and/or the inhibitory microenvironment.^68,81,100,101^

Early intranigral transplantation studies largely failed because long-distance axonal regrowth to the correct target(s) within the CNS is limited.^77,102,103^ Despite the development of various approaches to increase neurite outgrowth for targeted reinnervation, these strategies might result in the innervation of aberrant targets and/or only attain sub-centimeter scale growth, thus insufficient to reach what would be required in the human brain (i.e., substantia nigra to striatum is at least 3 cm).^76,96,104–106^ For example, a recent study reported that transplanation of human ventral mesencephalic patterened progenitor cells established extensive innervation towards appropriate forebrain targets and reduction of behavioral deficits in rodents.^80^ Although these findings are promising, off target growth is difficult to predict and/or prevent with increased distance between the graft and intended target, especially in larger animal models and humans.

However, a potentially transformative approach is being advanced to create axon guidance paths via factors secreted by host cells transfected with precise spatial precision (via stereotaxic microinjection) to overexpress growth factors, creating chemotaxic gradients to drive long-distance, targeted axonal outgrowth from endogenous and/or transplanted cells in the CNS.^107,108^ Although extremely promising, the scalability of this strategy to centimeter scale axon paths will be paramount, as clinical translation requires consistent and long-distance outgrowth with precise targeted reinnervation to restore the full motor circuit.

To address the challenging regenerative distance, early studies reported using embryonic striatal tissue as a “stepping stone” to effectively enable targeted outgrowth from axons grafted in the SNpc toward the graft, and ultimately the host striatum.^109^ Although this strategy appeared promising, in primate studies, the attraction was greatly diminished with increasing distance between the striatal “stepping stone” and the SNpc graft and the preferential attraction to the striatal “stepping stone” prevented outgrowth beyond the graft to the intended host striatum.^105^ Other “bridging” strategies have been proposed, such as fetal striatal tissue, fibroblast growth factor-4 secreting schwannoma cells, GDNF-secreting Schwann cells, and kidney tissue.^101^ Bridging the nigrostriatal pathway with growth factors or cells alters the inhibitory microenvironment and provides a supportive conduit for regenerating axons that effectively guides immature axons from the SNpc to the DA-depleted striatum.

Alternative strategies have been explored to improve targeted striatal reinnervation from fetal mesencephalic tissue grafted in the substantia nigra, such as injecting kainate between the midbrain and striatum to create a trophic environment for axonal outgrowth.^110^ Although kainate administration was shown to increase striatal dopamine release and reduced motor behavioral deficits, kainate is an excitatory neurotoxin, thus significantly limiting its potential regenerative strategy for patients with PD.^111^ Following this approach, a similar strategy has been investigated using GDNF as an attractant for guiding transplanted axons from the SNpc to the striatum. Administering a track of GDNF from the fetal graft in the SNpc to striatum facilitated outgrowth and improved rotational behavior in lesioned rats.^112^ This strategy was adapted in a NHP model of PD that utilized viral-vector-mediated overexpresion of GDNF in the striatum, enabling outgrowth from a fetal graft in the SNpc toward the striatum. In this study, only a few grafted dopaminergic axons innervated the striatum, however, these findings demonstrated neurotrophic supplementation could enable targeted long-distance axonal outgrowth.^106^

Although the ideal location for cell transplantation is likely the SNpc, successful striatal innervation in humans and NHPs requires significantly more-targeted axonal outgrowth to achieve a regenerative distance on the order of centimeters.^61,113^ Although rodent transplantation studies have reported some improvement in motor functionality, consistent recovery in humans would likely require greater striatal reinnervation.

A promising strategy for reconstruction of the nigrostriatal pathway utilizing multiple transplantations along the basal ganglia circuitry has been reported to enable complex movement patterns in models of PD.^103,114,115^ Extranigral transplants may provide trophic support to otherwise denervated host neurons, preventing widespread degeneration from second-order neuron neurotransmitter depletion, thereby increasing the number of potential targets for regenerating axons from dopaminergic neurons transplanted in the SNpc. Reinnervating the extranigral regions with dopaminergic neurons extending from the SNpc that receive proper local inputs from other cell populations likely closes the motor feedback loop, restoring the ability to generate complex movement patterns.^115^ In a small open-label pilot study, simultaneous nigral and striatal transplants resulted in significant long-term clinical improvement of motor function in patients.^116–118^ Although intriguing as a regenerative strategy for PD, further research is necessary to understand the exact mechanism behind the impressive functional recovery and graft survival in rodents and humans.

Another important consideration for development of cell-based strategy for PD is the potential for graft-induced dyskinesia (GID). Clinical trials with human embryonic tissue have demonstrated that dopaminergic neurons can reinnervate the striatum, release dopamine, and functionally integrate with the host brain.^65^ However, inconsistent functional recovery has been reported across multiple clinical trials: although patients experienced symptomatic relief ranging from nonexistent to significant improvement, ~15% of patients developed off-phase dyskinesia.^119^ GID is an adverse effect likely caused by runaway dopamine release and excessive outgrowth from the embryonic graft.^100^ Unlike the typical presentation of dyskinesias in patients with PD caused by chronic l-DOPA administration, GID-induced abnormal motor movements do not resolve with reduction and/or cessation of l-DOPA treatment.^120^ Clinical findings from these studies revealed an association between the development of GID and the preoperative presentation of l-DOPA-induced dyskinesia (LID); specifically there was a high incidence in patients who reported severe motor fluctuations prior to transplantation.^100^

It has been suggested that biological rejection and persistent low-grade inflammatory response might compromise the tissue graft, leading to neurotransmitter dysregulation and ultimately development of dyskinesias.^121^ This hypothesis originated from the clinical observation that GIDs were only reported in patients who either did not receive immunosuppression or only short-term, low-dose immunosuppression after fetal engraftment.^122^ Other studies have suggested the presence of serotonergic neurons in the graft might play a critical role in the expression of GIDs.^123^ The mechanism is still poorly understood, but it is likely that serotonergic neurons were transplanted in the graft, which led to a dysregulation of dopamine release. Indeed, in a clinical study without any patients who developed GID, the postmortem analysis revealed a 2:1 ratio of dopaminergic to serotonergic neurons in the graft regions.^118^ Future clinical trials for fetal engraftment might need to include immunosuppression and avoid patients with established LID to increase the likelihood for successful transplantation, a strategy that has been adopted by various ongoing and upcoming clinical trials.^124^

### Current state of clinical trials for PD

As described elsewhere in this review, early human fetal ventral mesencephalic transplantation studies demonstrated graft survival, reinnervation of the striatum, and functional restoration. However, it has been argued that the development of adverse events, such as GIDs, as well as failure of two double-blinded, placebo-controlled studies to show differences between patients transplanted with human fetal ventral mesencephalic tissue and control-treated patients led to diminished interest in cellular therapies for treatment of PD. In the last decade, thorough reanalysis of these clinical observations provided the basis for well-defined criteria in future clinical trials. This approach led to the establishment of a European Union-funded multi-center team (TRANSEURO) for a human ventral mesencephalic tissue transplantation study that has focused on the implementation of well-defined criteria, such as patient selection; tissue dissection, preparation, and storage; grafting technique; immunosuppression protocol; and experimental design. However, the TRANSEURO study was unable to complete enrollment owing to scarcity of human fetal tissue supply, leading to only 20 of the planned 90 patients undergoing transplantation.^124^

Human pluripotent stem cells (hPSCs) derived from ESC and iPSCs are an attractive alternative cell source that avoids the ethical and practical complications with fetal tissue. In 2014, different teams from academic institutions across Europe, the United States, and Japan began a new global initiative, GForce-PD, that aims to advance hPSC-derived dopaminergic neurons to first-in-human-clinical trials.^124^ Although human ESC-derived dopaminergic neurons likely have the capability to innervate the putamen, some teams have chosen to use HLA-matched, autologous iPSCs to avoid the need for immunosuppression.

### Tissue engineering: combining cells and scaffolds

The objective of the field of neural tissue engineering is to utilize biomaterial scaffolds and cell-based strategies in combination to augment endogenous regeneration and/or to provide direct replacement of neural cells and circuitry.^125^ Incorporated cell types may include primary, stem, differentiated, genetically engineered, autologous, allogeneic, or heterologous cells. Biomaterials utilized within the constructs often provide structure, protect cells (implanted and/or host), and produce an environment in which cells can adhere, migrate, differentiate, and signal to each other and to the host. Tissue-engineered constructs may possess a defined architecture that not only facilitates integration of the transplanted cells/processes with native tissue, but also maintains their desired organization.^126^ This architecture may be precisely engineered to match the structure and properties of the tissue for integration: to provide directionality for infiltration from implant to host, vice-versa, or both.^5,127–129^ Biomaterials may be synthesized to promote desired cellular organizations or mechanical properties (e.g., rigidity or elasticity) that are directionally dependent (anisotropic). Likewise, gradients of factors, such as growth factors and signaling molecules, may be used within tissue-engineered scaffolds to generate anisotropic features. The permissive microenvironment or proper matching of mechanical features created by some biomaterial hydrogels may also improve graft survival by influencing inflammatory reactions and minimizing the foreign body response.^130^

Previous research has indicated that long-distance axonal outgrowth rarely occurs in the mature nervous system, and therefore, to restore the full motor circuitry, alternative methods are needed to repair the long-distance circuits disrupted in PD. Biomaterial scaffolds are an alternative to facilitate long-distance axonal outgrowth as they can aid in reconstructing the nigrostriatal pathway by coaxing long-distance axonal outgrowth from the SNpc to appropriate targets in the striatum (either by endogenous or exogenous cells). Studies attempting to restore long-distance axonal connections typically aimed to create a permissive environment for axonal outgrowth, and/or augment the intrinsic capacity of axons to regenerate.^131,132^ These strategies most commonly involve biomaterial or cellular scaffolds that provide growth-promoting cues or reduce inhibitory factors.^133,134^ Although notable, on their own, biomaterial scaffolds do not address the degeneration of source neuronal population(s). Therefore, most scaffold approaches in PD are aimed at promoting a hospitable environment for implanted cells rather than outgrowth from endogenous neurons.

An emerging strategy in neural tissue engineering involves the development and application of so-called “living scaffolds”, which are defined as constructs with a preformed, often anisotropic architecture, consisting of living neural cells within a 3-D biomaterial matrix.^5,126,129,135^ In particular, our group is pursuing the creation of tissue-engineered “living scaffolds” for several applications, including to provide regenerative pathways for axonal guidance or neuronal migration, and also to directly replace neurons and axon tracts in order to reconstruct degenerated neural circuits.^5,125,126,128,129,135–137^ Most relevant to PD, tailored constructs may be developed to structurally and functionally emulate the nigrostriatal pathway, toward the goal of replacing dopaminergic neurons and their long axon tracts. This strategy is premised on the plasticity of endogenous as well as tissue-engineered neural networks, whereby neurons intrinsically have the ability to sense and respond to local activity.^138,139^ It has been shown that transplanted neurons are capable of receiving synaptic input from local networks as well as propagating action potentials.^140^ Once the appropriate synapses are established, preformed anatomically inspired constructs could act as functional relays to transmit signals between populations of previously disconnected cells.

**Fig. 4:**
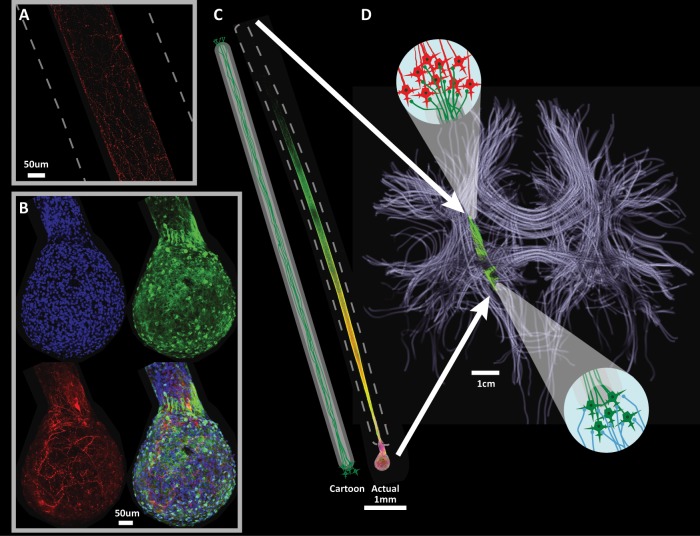
Reconstruction of the nigrostriatal pathway using microtissue-engineered neural networks (micro-TENNs). **a** Immunocytochemical image of the axonal segment of a micro-TENN showing the robust outgrowth of dopaminergic axons in **c**, as labeled using an antibody for tyrosine hydroxylase (TH; red). The hydrogel shell is highlighted with a dotted line. **b** Immunocytochemical image of the somatic end of a uniaxial micro-TENN showing a large cluster of aggregated neurons in **c**, labeled with a Hoechst nuclear counterstain (blue) and using antibodies for all neurons/axons (β-tubulin III; green) and dopaminergic neurons/neurites (TH; red), with an overlay of all three. **c** The cartoon (left) and actual (right) unidirectional micro-TENN show the long-distance axonal outgrowth. The bolus of neurons is at the bottom with axonal outgrowth projecting upwards. The actual micro-TENN has the same staining as **b**, and the hydrogel shell is highlighted with a dotted line. **d** A diffusion tensor imaging representation of the long-distance axonal tracts (lilac) that connect discrete populations of neurons in the human brain. This conceptual rendition shows how a unidirectional micro-TENN—consisting of a population of dopaminergic neurons extending long, aligned processes—can be used to recreate the nigrostriatal pathway (green) that degenerates in PD. The magnification inset in the lower right depicts axons (blue) in the substantia nigra functionally integrating with the transplanted dopaminergic neurons in the micro-TENN (green). The magnification inset in the upper left depicts transplanted dopaminergic axons (green) functionally integrating with neurons in the striatum (red). The micro-TENN implant theoretically recreates the full motor feedback circuit by receiving the stereotypical inputs in the SNpc while projecting axons to the striatum to release regulated amounts of dopamine in that structure.

As such, we are pursuing a novel regenerative medicine solution whereby custom-built microtissue-engineered neural networks (TENNs) are transplanted to physically replace SNpc neurons and their long-distance axonal connections from the SNpc to the striatum (Fig. 4). Micro-TENNs are precisely formed, miniature constructs designed to mimic the systems-level architecture of the nigrostriatal pathway: a discrete population of dopaminergic neurons (Fig. 4b) extending long unidirectional axonal tracts (Fig. 4a) within hydrogel micro-columns (Fig. 4c, d). This tissue engineering-based strategy provides several advantages. These constructs are fully grown in vitro prior to in vivo implantation, allowing for verification of neuronal-axonal architecture, neuronal health, axonal projection length, and dopamine production/release, as well as providing an opportunity to screen for unwanted characteristics such as the presence of undifferentiated and/or oncogenic cells in the case of stem cell-derived constructs. The columnar hydrogel encasement initially ensures the formation of the desired architecture during growth in vitro while providing physical protection of the engineered neuronal networks and axonal tracts during implantation and degrading over weeks to gradually introduce the majority of the construct surface area to the brain. This physical protection coupled with the implantation of not only neurons and axons but their 3-D microenvironment as well, may minimize neuronal/axonal loss during transplant and promote survival and integration post transplant.

Therefore, micro-TENNs may have the capacity to restore the nigrostriatal circuitry lost to PD by replacing the dopaminergic neurons in the SNpc, enabling synaptic integration with inputs to the SNpc, and restoring axonal terminals in the striatum. Importantly, pathway reconstruction approaches such as the micro-TENNs have the promise to restore the aspects of the motor feedback path, a feat not possible with methods such as pharmacological dopamine replacement therapy, DBS, or tissue grafts in the striatum. As such, we are currently assessing the ability of dopaminergic micro-TENNs to replace SNpc neurons and restore dopaminergic inputs to the striatum in rat models. In recent proof-a-concept study, microtissue-engineered neural constructs containing a population of dopaminergic neurons on one end and long axonal projections grown through a hydrogel column were fabricated, mimicking the entire nigrostriatal pathway.^141^ Cell source and construct length were evaluated and evoked dopamine release was confirmed in vitro. These constructs were stereotactically implanted en masse to mimic the nigrostriatal pathway in a rodent model. At 1-month post implant, histological data revealed neuronal survival and maintenance of axonal architecture. Further studies investigating the synaptic integration and amelioration of motor deficits in a rodent model of PD are ongoing. If successful, dopaminergic micro-TENNs will be the first strategy to facilitate nervous system repair by simultaneously providing neuronal replacement and physically re-creating long-distance axonal connections in the brain. In addition, we are also establishing the future potential of personalized micro-TENNs—built using neurons derived from a patient’s own stem cells—as a potential solution to restore the dopaminergic nigrostriatal pathway and ameliorate motor symptoms without the need for immunosuppression.

As discussed in previous sections, treatment of many non-motor symptoms of PD has remained challenging owing to the widespread dysregulation of the circuitry connecting basal ganglia, prefrontal cortex, and limbic systems.^15,19^ To date, micro-­TENNs have been fabricated using dorsal root ganglia neurons, cerebral cortical neurons (e.g., mixed glutamatergic and GABAergic), embryonic rodent ventral mesencephalic dopaminergic neurons, and human ESC-derived dopaminergic neurons.^5,125,126,129,141–144^ As our understanding of PD pathophysiology expands, it is possible that multiple micro-TENNs could be transplanted to reconnect different damaged regions, comprised of alternative neuronal phenotypes, such as noradrenergic, serotoninergic, and cholinergic cell types. However, it is difficult to predict how transplant therapies, including either transplanted cells or pathways, such as micro-TENNs, could be used to recapitulate widely dispersed innervation of cerebral cortex from cholinergic, serotonergic, or noradrenergic brainstem nuclei.

### Cell engineering and reprogramming

Promising recent work has utilized viral vectors to re-program endogenous non-neuronal cells to become neurons.^145^ This approach generally alters the gene expression of astrocytes—an abundant glial cell type in the area of degeneration—to convert them into neurons as a means for localized neuronal replacement. Direct reprogramming of striatal astrocytes to dopaminergic neurons in a rodent model of PD has been reported to improve dopamine tone and reduce motor deficits.^94^ Although extremely promising, conversion of striatal astrocytes would not reproduce the nigrostriatal circuit, and conversion of nigral astrocytes would require coupling with an axonal pathfinding strategy as described above to elicit target connections to the striatum.

Cellular engineering strategies may also be advantageous for cell transplants, with such genetic manipulation being employed in vitro before in vivo implantation. For instance, specific genetic alterations may be beneficial to improve survival, integration, and efficacy. One type of modification could be to limit immunogenicity and thereby improve the survival and integration of the cells.^146^ However, cell-based or tissue-engineered constructs (either allogeneic or autologous) may eventually succumb to the underlying pathology and degenerate similar to native tissue. Indeed, recent studies have hypothesized that dysfunction of iron metabolism in the substantia nigra might be implicated in the degeneration of dopaminergic neurons and ultimately the progression of PD.^147^ Although the susceptibility of transplanted cellular constructs to the typical progression of PD pathology is still unclear, dopaminergic neurons from tissue grafts can survive up to 24 years in vivo despite ongoing degeneration of the native dopaminergic system.^52^ However, in some patients with long-term survival of fetal mesencephalic grafts, α-synuclein-positive Lewy bodies were found in 1–5% of transplanted dopaminergic neurons at 12–22 years and 12% in one patient at 24 years post transplantation. Thus, the development of PD-resistant neurons might be necessary to overcome this vulnerability, improve the construct lifespan, and prevent α-synuclein aggregation by augmenting the genes implicated in autophagy, α-synuclein clearance, or decreased iron accumulation in the substantia nigra.^148,149^ Furthermore, genetically enhanced dopaminergic neurons engineered to increase dopamine production and/or release could minimize the number of surviving cells necessary to restore dopamine levels in the striatum, thus maximizing the likelihood for successful innervation of the striatum, and potentially halting the evolution of PD symptoms.

## Closing: challenges and opportunities

The classic motor symptoms of PD result from selective degeneration of dopaminergic neurons in the SNpc, and subsequently the disruption of key motor control circuits. This circuit disruption results from loss of the finely tuned dopamine delivery to the striatum via long-distance axonal projections. Current treatments for PD, including the use of dopamine replacement strategies and DBS, are aimed at treating symptoms rather than the underlying neurodegeneration. Newer treatments have focused on neuroprotection, but to date, no therapies have been shown to clearly slow progression of neuronal degeneration and hence motor symptoms.^10,38^ In addition, dopaminergic input to the striatum requires continuous modulation and feedback from other neural structures via SNpc inputs to alleviate potential runaway dopamine excess and dystonia, a potential side effect from cell transplants into the striatum.

To address these gaps in treatment, emerging regenerative medicine solutions are being pursued to replace lost neurons and axonal circuitry in PD. Indeed, the ultimate goal is to replace the nigrostriatal pathway—dopaminergic neurons in the SNpc and their axonal projections to the striatum—thereby allowing implanted cells/tissue to be subject to the normal cellular regulation that dopaminergic SNpc neurons are subject to in order to “close the loop” and restore this crucial circuit for motor control feedback. Reconstructing the pathway from the SNpc to the striatum is the most likely means to provide dopaminergic inputs that can be tuned and controlled by natural feedback mechanisms present within the brain. On this front, our research team is pursuing a tissue engineering-based strategy to accomplish this goal, as we are developing preformed, implantable column-like micro-constructs that mimic the architecture of the nigrostriatal pathway: a discrete population of dopaminergic neurons with unidirectional, long-projecting axonal tracts. The method may be uniquely suited to simultaneously replace lost dopaminergic neurons within the SNpc and recapitulate the full nigrostriatal pathway—spanning several centimeters—to provide naturally regulated dopaminergic inputs to the striatum. Unlike DBS, which attempts to disrupt pathologic activity in the indirect pathway, our preformed micro-constructs are themselves an auxiliary pathway. In general, such precisely engineered constructs may be highly controllable, where the number of neurons and generation of dopamine can be known prior to implantation, thus potentially ameliorating the inconsistency historically seen in fetal tissue grafts.

In order to supply the requisite number of healthy dopaminergic neurons needed for functional improvement in humans (estimated to be 40,000–80,000 neurons), cell sources will need to be significantly scaled up prior to clinical trials. Stem cells and other expandable, self-renewing cell sources—whether allogeneic or autologous—are promising to address these needs. Such methods, while presenting challenges in controlling differentiation, heterogeneity, and maturity, among others, will avoid the limitations of fetal tissue grafts, specifically ethical considerations and dearth of fetal source material. In particular, using emerging iPSC methodology, a patient’s own cells could be efficiently differentiated into dopaminergic neurons, thereby eliminating the need for immunosuppression.^82^ Combined with tissue engineering methodology, such autologous stem cell sources may enable the construction of patient-specific constructs tailored to their particular extent of degeneration. Such next-generation constructs may provide a transformative and scalable solution to directly replace SNpc neurons, restore axon-based dopaminergic levels in the striatum, and thereby alleviating the cause of motor symptoms in PD. However, similar to the current limitations to treatments for PD, potential benefits for cell-based neurorestorative strategies would likely be dependent on the duration of disease and thus degree of neurodegeneration. In the coming era of restorative neurosurgery, emerging regenerative medicine therapies may revolutionize PD treatment by permanently reconstructing lost neuroanatomy and markedly improve outcomes for millions of patients worldwide afflicted by PD.

## Data Availability

Data sharing not applicable to this article as no data sets were generated or analyzed during the current study.
